# An investigation of the use of rectangular insecticide-treated nets for malaria control in Chipinge District, Zimbabwe: a descriptive study

**Published:** 2012-09-06

**Authors:** Shadreck Sande, Paul Jagals, Bartholomew Mupeta, Addmore Chadambuka

**Affiliations:** 1 Tshwane University of Technology, Department of Environmental Health Private Bag X680 Pretoria 0001, South Africa; 2 Plan Zimbabwe Private Bag HG 7232 Highlands Harare, Zimbabwe; 3 Ministry of Health and Child Welfare, P.O. Box CY 1122, Causeway, Harare, Zimbabwe

**Keywords:** Insecticide treated nets, rectangular insecticide treated nets, triangulation, mounting, senior matriarchs, net use

## Abstract

**Introduction:**

In 2007, Zimbabwe government distributed rectangular insecticide treated nets in Chipinge District, covering 100% of population at risk. However, malaria morbidity continued increasing from 492/1000 (49.2%) in 2007 to 667/1000 (66.7%) in 2008. A study was conducted in Chipinge District in May 2009 to investigate the use of rectangular insecticide treated nets and factors affecting their use in malaria prevention.

**Methods:**

A descriptive cross-sectional study was conducted. Quantitative and qualitative methods were triangulated to assess utilisation of rectangular insecticide treated nets. Five interviewers administered 380 questionnaires to senior matriarchs selected from five wards, with 19,667 sampling frame (19,667/380 = 52). Five focus group discussions were conducted. Quantitative data were analysed using Statistical Package for the Social Sciences, while qualitative data were summarised into thematic areas.

**Results:**

Approximately, 95% of respondents knew that malaria was caused by mosquito bites. Perception of nets as malaria preventative measure was high (88%). Utilization of rectangular insecticide treated nets was low (33%) with 81% of those not using them expressed difficulty procedures of mounting them and unavailability of related accessories as main reasons. People preferred conical insecticide treated nets (84%) compared to rectangular insecticide treated nets (15%).

**Conclusion:**

Although the Chipinge people accepted insecticide treated nets for malaria prevention, procedure of mounting rectangular insecticide treated nets and accessing related accessories prevented consistent use.In order for insecticide treated net project to have impact on malaria prevention, priority should be given to conical shape or rectangular shape with adequate accessories like wire nails and strings or twine.

## Introduction

The global incidence of malaria is substantial, with an estimated 300 million suspected cases each year of which one million die. More than 90% of these deaths occur in sub-Saharan Africa where young children are the most affected [1]. Malaria is a serious public health problem, causing suffering, deaths and poverty in Zimbabwe [2]. It is the third commonest cause of morbidity and mortality, coming after HIV and AIDS and tuberculosis across all age groups, with around 1.5 million malaria cases and approximately 1,000 deaths occurring annually over the past five years [2].

Several measures which included use of insecticide- treated nets, indoor residual spraying (IRS), larviciding, environmental management, intermittent preventive treatment in pregnancy and community education were put into action to control both the malaria vectors and parasites, to reduce malaria cases in Zimbabwe.

**Figure 1:**
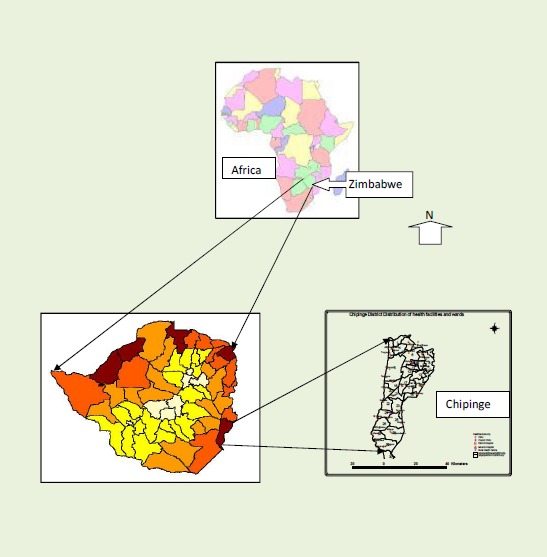
Geographical location of Chipinge district in Zimbabwe

The Zimbabwe government distributed free insecticide-treated nets for malaria prevention in Chipinge district. The district is located in Manicaland Province and is situated in the south eastern part of the country (Figure 1). Among the available malaria interventions, use of insecticide-treated nets (ITN) finally became the major intervention to limit malaria incidence in the district which is located in the high malaria transmission zone in Zimbabwe. ITN availability was scaled up by way of their free distribution, achieving near 100% coverage of the population at risk of contracting malaria.

The local ITN intervention strategy in Chipinge was to distribute at least one insecticide-treated mosquito net per two people for all households located in ten municipal wards, on so doing targeting a level of protection of about 100% of the population at risk of malaria. The distribution of free ITN in Chipinge District was meant to support the WHO's recommendation for universal access to ITN where all people living in malaria affected areas were expected to sleep under ITN every night all year round [1].

Insecticide-treated nets were proven to have a powerful impact on reduction of vector-borne diseases including malaria when used correctly and consistently [3–5]. Of particular importance was the users’ choice of mosquito net in terms of shape and colour as well as practice to mend them when damaged [4]. This was a major problem in the use of, especially the rectangular ITN.

Rectangular mosquito nets are often used where malaria or other insect-borne diseases as well as nuisances are common, especially as a tent-like covering over a sleeping bed. For effectiveness (the potential impact of an intervention, when deployed in real life conditions), the mosquito net must not rest directly on the skin of the sleeping person because mosquitoes can then reach and bite a person through the mosquito net webbing. When they are hung over sleeping beds, rectangular mosquito nets provide more room to the user than conical nets. Rectangular ITN have been used in several countries in Africa such as Tanzania and Nigeria and their acceptance was good. Evidence from these countries has shown that some rooms in the households were potential sleeping areas which would warrant hanging rectangular ITN during bed time and removing in the morning [6, 7].

Of the available ITN shapes on the market, the people of Chipinge were introduced to rectangular as well as conical ITN for use in homes to prevent malaria. Conical shaped ITN were introduced before rectangular ones which implied that the community in Chipinge became used to conical rather than rectangular ITN by the time the latter were introduced ten years later. The mosquito net industries designed and manufactured both rectangular and conical ITN for malaria prevention at homes. The rectangular ITN was introduced to achieve what conical often could not as it reduces body contact between the user and the mosquito net; all in all initially seen as improvements in terms of ITN as a malaria prevention tool. Despite 100% ITN coverage in Chipinge, malaria incidence continued to increase from 49.2% in 2007 to 66.7% in 2008 [8]. Based on the assumption that effective use of ITN in an area with 100% coverage should reduce malaria incidence; it was not clear why this efficiency was not also achieved in the Chipinge district. This study investigated the use of rectangular insecticide treated nets (ITN) and factors affecting their use in malaria control.

## Methods

A descriptive cross-sectional study using a triangulation strategy (combining quantitative and qualitative methods) was conducted in Chipinge District in May 2009. A major reason for combining research methods (quantitative and qualitative) in this study was to ensure that the findings from one method are elaborated, or clarified by the findings of the other method [9].

Chipinge District is divided into ten wards (clusters) based on local government administrative boundaries and random sampling was used to select wards. Using rule of thumb, 50% of the total number of wards was randomly selected by numbering wards from one to ten. Five wards were sampled using the random number selection tool in Microsoft Excel^®^. A sampling frame for each ward was drawn from the list of households that were issued with ITN in the district. Systematic sampling was conducted to select the required number of households in each ward using information obtained from master ITN distribution register (kept at Chipinge District Hospital) i.e. depending on sample size (n) and the total population in the ward, every 52nd household was selected to be part of the sample.

The respondents for both households and focus group discussions (FGD) were systematically selected as every result of all variables in this study relied strongly on the opinion or status of the respondents. For the household questionnaire, household heads or their spouses (senior matriarch) were singled out and interviewed. Participants for FGD were village community workers (VCW) who were also members of the households in the study area. The VCW were randomly selected from VCW master register kept at Chipinge District hospital and were invited for FGD. Village community workers were selected to participate during focus group discussions on the basis that they were the Chipinge community volunteers chosen by the community to promote public health, hence their opinion and status were thought to well represent the community of Chipinge District.

The study population was all Chipinge households that were issued with ITN. A total of 59000 ITN were distributed in Chipinge district. Each household received an average of three ITN, (based on six members per household). The population under study (N) was 19667 households.

The sample size (n) required to adequately represent the population (N) in this study was estimated using a formula according to Saunders et. al. (2009), assuming 95% confidence level and maximum allowable error at 5%, the estimated sample size was 365 households. It was adjusted to 380 assuming a response rate of 95%.

Households’ heads or their spouse (senior matriarch) who were present at the chosen households at the time of data collection were purposively recruited in the study by the interviewer and were interviewed. Five focus group discussions (FGD); one for each ward, were conducted. The participants were village community workers who were also members of the households in the study area. FGD were conducted at a rural health centre situated in each ward. The number of FGD was kept at five because information obtained after the fifth FGD was assumed would not any longer be new or would seem repetitive [9].

Quantitative data were analyzed using Statistical Package for the Social Sciences (SPSS) Version 10.0. Data were entered and cleaned before analysis. Frequency, percentage distribution tables and cross tabulation were part of the data analysis. Focused group discussion data were analysed by summarising and simplifying and/or selectively focusing on some parts of the data and interpretation was done.

Ethical approval to conduct the research was obtained from Tshwane University of Technology Research Ethics Committee and permission from relevant government structures and community representatives. Confidentiality was assured to the participants and a signed informed consent form was obtained before each participant was interviewed in the study.

## Results

### Demographic characteristics of the study participants

Two hundred and eighty eight (76%) of the respondents were females and 91 (24%) were males. The median age was 28 years (Q1 = 22 years, Q_3_ = 37 years). Two hundred and thirty seven (85%) were married, 10 (4%) were single and the remainder were either widowed or divorced. More than half (85%) of the respondents had at least attained primary education which might imply that the community in Chipinge could easily understand and implement new technology like consistent use of rectangular insecticide treated nets (rITN).

### Knowledge of the participants on malaria

Respondents indicated two causes of malaria (Table 1), of which 95% (254/266) understood that malaria was caused by mosquito bites. To the multi-response question to determine the knowledge levels on symptoms of malaria, respondents answered fever, chills, headache, vomiting/diarrhoea and joint pains (Table 2). Of all the symptoms of malaria mentioned, fever, chills and headache were collectively mentioned by most of the respondents (39.7%).

**Table 1:** Community perception on the causes of malaria in Chipinge District, 2009

Causes	Frequency
Mosquito bites	254 (95.5)
Dirty water or food	10 (3.8%)
Do not know	2 (0.8%)
Total	266 (100%)

**Table 2:** Knowledge of signs and symptoms of malaria among adults in Chipinge District, 2009

Signs and symptoms	Frequency
Fever, chills and headache	150 (39.7%)
Fever, chills and vomiting/diarrhoea	35 (9.3%)
Fever, headache and vomiting/diarrhoea	61 (16.1%)
Chills, headache and vomiting/diarrhoea	65 (17.2%)
Chills, headache and joint pains	39 (10.3%)
Headache, vomiting/diarrhoea and joint pains	28 (7.4%)
**Total**	**378 (100%)**

### Seasonal variations in mosquito density

The community perceived that mosquito breeding varied by season. The majority of respondents (79.6%) observed the highest mosquito denisty (number of mosquitoes in a given area) during the rainy season. Slightly more than 15% of the population indicated that they observed the greatest mosquito density during the dry season.

### Distances between homesteads and mosquito breeding sites

The respondents’ perceived the distances between mosquito breeding sites and their homesteads to be between 100 to 1,000 metres. Majority of the respondents (44.3%) revealed that the major mosquito breeding sites were within 100 metres. Eighty seven percent indicated that they resided within 1,000 metre (one kilometre) radius away from mosquito breeding sites.

### Households issued with ITN

Most households (93%) confirmed having been given rectangular ITN by Zimbabwe government in 2007 while 7% said they were not given mosquito nets at all. However, the ITN coverage of 93% is high compared with World Health Organization guideline of acceptable ITN coverage of at least 80% [1]. According to this guide, for ITN intervention to make an impact in malaria prevention, the coverage should be at least 80%.

### Insecticide-treated nets (ITN) use for malaria prevention

Thirty three percent (n = 372) of the respondents reported to have slept under insecticide-treated nets the previous night; whilst larger percentage 67% (n = 372) did not. ITN coverage is not synonymous to use as the reported coverage was 93% and use 33% (Table 3).

**Table 3:** Reported insecticide-treated net use for malaria prevention in Chipinge District, Zimbabwe, 2009

ITN use the previous night	Frequency
Yes	124 (33%)
No	248 (67%)
**Total**	**372 (100%)**

### Number of sleeping beds/mats under insecticide-treated net

The total number of sleeping beds/mats for the 379 households in the five clusters was observed to be 1,139, giving an average of three sleeping beds/mats per household. Of these sleeping beds/mats, only 30.8% were observed to have ITN hung over them.

### Knowledge on hanging insecticide-treated nets (ITN)

Eight two percent of the respondents reported having been trained on hanging ITN by health workers and knew how to hang them. Of those respondents who knew how to hang ITN; 89% demonstrated the correct hanging procedures.

### Major problems related to consistent use of rectangular ITN

The main reason for not hanging ITN over sleeping beds/mats was that the procedures were very cumbersome for the majority (81%) of the participating households (Table 4).

**Table 4:** Reasons for failure to hang rectangular insecticide-treated nets (rITN) over sleeping beds/mats in Chipinge District, Zimbabwe, 2009

Reason	Frequency
ITN not enough	15 (4.1%)
Rooms too small to fit rITN	26 (7.1%)
Fitting rITN is very difficult	2998 (81.0%)
ITN make people suffocate	8 (2.1%)
Fitting of ITN is done at sleeping time and removed early morning	10 (2.7%)
Other	11 (3.0%)
**Total**	**368 (100%)**

### Seasonal use of ITN

Most respondents indicated that they mostly use ITNs for the eight summer months of the year (September to April). During these months, ITN use generally exceeds 50% and this becomes considerably less than 50% during the winter starting May up to August. The months during the course of the year which had the lowest and highest ITN usage were June (13%) and January (84%) respectively coinciding with times perceived by the community to be of lowest and highest level of mosquito density. The peak period for malaria transmission in Zimbabwe in general, Chipinge District in particular is in April [6]. The present study has shown ITN usage for April to be 52%, which is 28% short of the WHO guidelines (80%). This implies that during malaria transmission peak in Chipinge (April), only 52% of the people at risk of malaria are protected by ITN.

### ITN shape preferences of the participating households

The households in the study area preferred conical (84.2%), then rectangular (15.3%) with the square ITN making up a negligible 0.5% (Table 5).

**Table 5:** insecticide-treated nets (ITN) shape preferences for study participants in Chipinge District, 2009

Shape	Frequency
Rectangular ITN	58 (15.3%)
Conical ITN	319 (84.2%)
Other (square)	2 (0.5%)
**Total**	**379 (100%)**

### Perceptions of the relationship between ITN and preventing malaria

Eighty eight percent (242/275) of the households perceived ITN as a preventive measure for malaria while the remainder did not have this perception.

### Perceived extra benefits associated with rectangular insecticide-treated nets (rITN)

Most respondents (60%) indicated that the rITN offer no extra benefits over any of the other ITN shapes that communities were using. Thirty four percent were happy with the square fit to sleeping beds/mats by the rITN. Six percent indicated other extra benefits such as less contact between user and mosquito net.

### Households’ suggestions for modifications to rectangular insecticide-treated nets

Eighty six percent of participating households could not think of any modifications that could be done on the rITN in order to improve its ease-of-use, whereas 14% indicated that rITN could be modified by providing a single bracket at the centre of the top closed part so that it could be hung in the same way as conical mosquito net which is only hung on one position which is found at the centre of the top end. The extent to which the study community believed that the modifications of rITN were applicable in their homes or whether the modifications mentioned were feasible or were simply abstract issues was not explored further.

### General acceptability of insecticide-treated nets (ITN) for use by household

Ninety five percent of the respondents accept ITN for malaria prevention in their households, implying that the people are prepared to protect themselves from malaria. What seemed to be evident from these findings is that whilst people accepted ITN, the cumbersomeness of hanging rectangular ITN seemed to be a setback.

### Focus Group Discussion Findings

#### Knowledge

The responses showed that the community generally knew that malaria is caused by mosquito bites as the following quote exemplifies: *“Mosquitoes feed on human blood and malaria is transmitted by mosquito bites in the process of feeding.”* (FGD Participant)

FGD participants knew the signs and symptoms of malaria and came to a consensus that they also understood that the elderly, children less than 5 years old, pregnant women, people living with HIV and AIDS were more vulnerable of malaria mainly due to their low immunity. As a result, participants strongly pointed out that implementation of malaria intervention- use of insecticide-treated nets (ITN) for example; the vulnerable groups should have high priority. Hanging ITN over beds/mats was correctly demonstrated by members participating in the FGD, confirming that rectangular ITN recipients were trained on hanging procedures.

### Major problems related to consistent use of rectangular ITN

Reasons for not sleeping under ITN or hanging ITN over sleeping beds/mats were further explored. The following quote from the FGD illustrates an important fact: *“My children listen (referring to facilitators and the moderator). Hanging your ‘rectangular mosquito nets’ is very difficult as one (rectangular mosquito net user) needs strings and wire nails. In Chipinge we (our family) are poor and do not have strings and wire nails which are suitable for hanging rectangular mosquito nets. The Government (Zimbabwe government) should give us (Chipinge community) the rectangular mosquito nets together with their accessories (strings and wire nails) like what used to happen when they (Zimbabwe government) gave us conical mosquito nets some years ago which came together with the rings.”*

### ITN shape preferences of the participating households

Preference of conical ITN was confirmed in all five FGDs who expressed that they were more comfortable in conical ITN because they were easy to hang. *“When using conical mosquito net, a person needs only one hanging position whereas in using a rectangular mosquito net, several wire nails and strings are required; which may not be available.”*

### Perceptions of the relationship between ITN and preventing malaria

The results of household survey were confirmed during FGDs where participants went a step further saying mosquito nets prevent malaria when used consistently by many people. *“All types of mosquito nets prevent malaria, particularly if all people use them every night.”* (FGD Participant)

### Perceived extra benefits associated with rectangular insecticide-treated nets (rITN)

Focus group participants were mostly noncommittal about any extra benefits associated with rITN, saying, only those who had realised the extra benefits should comment. *“I (participant) have not yet observed the extra benefits, perhaps someone (any focus group participant) can explain to me.”* (FGD Participant)

### Households’ suggestions for modifications to rectangular insecticide-treated nets

One participant in focus group who had made some modifications on rITN concurred that rITN can be modified to overcome the complexities of hanging: *“Parents (addressing focus group participants), allow me to share with you what modifications I have done to rITN in my own home. I got old wire rings which are used for cITN and tied them at the top closed end of the rITN to form a circle. I then added a bracket made of cloth at the centre of this closed end of the rITN.”* (FGD Participant)

When this modification was suggested in three other focus groups, participants did not understand the logic behind modification of rITN: *“Why should we (Chipinge community) modify a newly made mosquito net? If its application is not user-friendly, why bother, leave it alone.”* (FGD Participant)

### General acceptability of insecticide-treated nets (ITN) for use by household

The fact that ITNs are acceptable in Chipinge was evident in all focus groups but use of the rectangular ITN was presenting challenges. *“When the government distributed rectangular insecticide treated nets (rITN) in Chipinge, we (Chipinge community) all happily received them and took them to our homes for malaria prevention. We then had difficulties in hanging them every night. Surely the ITN are acceptable in Chipinge, what needs to be addressed is the hanging aspects of especially rITN which have troubled us (Chipinge community) a lot.”* (FGD Participants)

## Discussion

The study has highlighted several findings and recommendations which might promote consistent use of rITN for malaria prevention in Chipinge District. Our study population had a high literacy level with 85% (322/379) being able to read and write which was supposed to be an asset for rITN use but this was not so. The Chipinge community may have felt that government of Zimbabwe removed conical ITN from them for no apparent reasons and imposed difficult to use rectangular ITN. Using such high literacy level, the community of Chipinge accepted and took the rITN; perhaps due to fear of ‘Government victimization’ and did not consistently use them as was expected.

Furthermore, the study documented local knowledge and practices relating to malaria, its transmission and preventive measures. An important finding in this context was that people knew signs and symptoms of malaria and the methods which could be used to prevent malaria at local levels. This has consequences not only for treatment seeking behaviour as described in other studies [10] but also for prevention.

People's queries, critical reflections, arguments and active participation during focus group discussions and questionnaire administration suggested that they were seeking more knowledge on malaria prevention and control. If knowledge is seen as an ongoing interpretive practice which is hindered in process of social interaction and communication, community education on the use of ITN for example to prevent malaria, can therefore be carefully tailored to capitalize on these insights [11, 12].

For Chipinge ITN project, people might have viewed protection from mosquito bites in terms of avoiding nuisance biting, rather than preventing disease.Consequently, ITN were perceived as tools to protect the people from the biting of nuisance mosquitoes at night thereby affording them a better sleep. In such circumstances, using ITN every night all year round becomes more unlikely as the biting of a nuisance mosquito is less frightening than contracting malaria- which is associated with loss of production time, money and even in some instances- deaths [13–15].

Anopheles mosquitoes are usually found in large numbers less than three kilometers away from their breeding places [16]. An important finding in this study was that generally; majority of the people of Chipinge reside within mosquito flight range (less than 3 kilometres away from mosquito breeding places) which exposes them not only to malaria, but also to sleepless nights due to nuisance mosquito bites.

While efficacy of ITN (defined as the number of malaria cases or deaths prevented by an ITN intervention in a given population) can be calculated from randomized trials, it remains a challenge to translate efficacy recorded under trial setting into sustained effectiveness under program conditions [17]. More research is required on the effectiveness of ITN under real life settings where political, cultural, social and economic factors positively or negatively influence proper and consistent use of ITN [11, 12, 16].

The seasonal usage of ITN (lowest in June at 13% ad highest in January at 84%) by the people of Chipinge is a challenge which has not yet been resolved and may not be resolved with change of ITN technology such as shape [17]. Communities should be informed that even one infected female anopheles mosquito can cause malaria or death [18]. Therefore, it has been suggested that reinforcement of any factor that motivates people to sleep under ITN, even when it is not directly related to health, may help in trying to win people over to public health awareness and action [4, 19, 20].

Although privacy was found to be a motivating factor for ITN usage in studies in Ghana [21], it did not feature prominently in this study area, and may not be a good promotional indicator for this population. This could be attributed to the fact that in Chipinge, it was highly unlikely that one room had two or more sleeping spaces.

The use of rITN in the study population was quite contrary to previous indications from formative research that people desired to consistently use rITN but were only mainly barred by financial constraints [22–24].

The observed community reaction by low usage of ITNs (33%) raises questions about programs placing too much emphasis on action to the extent that implementers fail to recognize the processes of behaviour change.Findings from this study showed typical attempts to introduce new technology in the form of rITN without exploring how best to speed up their consistent use for malaria control to be achieved. The uncertainty inherent in new ideas is of course a concern, and a major determinant of people's evaluation of a new idea against previously existing alternatives [14]: *“We (our family) are waiting to see how the cumbersomeness of hanging them (rITN) shall be resolved by those who have started using them.”* (Excerpt from informal conversations heard while making rounds in the study community).

As in the Chipinge case, people would first need to acquire a new perception, that different shapes of ITN are hung differently and that it is, therefore, important to regularly use ITN in whatever shape with the same objective of malaria prevention.

Concerns over the daily hanging of ITN are more likely to bear important implications for consistent usage. Insecticide-treated nets in sleeping spaces including the living rooms and kitchens would definitely have to be put up and taken down again daily, both for neatness's sake and for safety against theft. While study population appears receptive about getting ITN, daily hanging remains laborious; prohibiting daily use of rITN as a malaria prevention tool.

How the uncertainty involved is addressed is of crucial importance.Health promotion plays an important role in predisposing people to malaria intervention. However, the relationship between knowledge and behaviour is not linear [12]. As in Chipinge case, people would first need to acquire a new perception that the rectangular ITN can be used just like conical ITN with an ultimate goal of malaria prevention. It is arguable that what was observed in this study was not necessarily failure, but rather a stage in the process of adopting the new innovation, raising questions of how to overcome negative influences while enhancing positive factors.

Results from this study clearly show that just increasing coverage (93%) will not be good enough. Unless people use ITN correctly and consistently, rates of malaria illnesses and deaths will not be reduced. Changing norms, behaviours, attitudes, practices and receptivity of new technology- like rITN, will be critical to increase mosquito net use. This will require more effective approaches by Zimbabwe government and its partners in terms of public education, promotion and marketing of new products- rectangular ITN included.

All in all, the results suggest high potential for the acceptance and use of ITN for malaria control in Chipinge provided that the cumbersomeness of hanging rITN is addressed. In addition, the apparent absence of any cultural barriers to ITN use is an added advantage in the future advocacy for insecticide-treated nets (ITN) in general.

## Conclusion

The effectiveness of ITN interventions on malaria depends to a large extent on human behaviour as a factor in health, influenced by socio-cultural traditions as well as economic and environmental determinants [25].Even though people's knowledge about relationship between use of ITN and protection from malaria was high, the usage was low. Fear of mosquito bites was the main motivation for sleeping under rITN. The respondents appeared to prefer cITN. The low preference of rITN was attributed to the cumbersome process required in mounting them; and also the inaccessibility of accessories such as wire nails and strings. Non reduction in malaria cases in Chipinge district after the implementation of ITN project as a major malaria intervention could therefore be attributed to ineffective use of ITN. In order for the ITN project to make impact in Chipinge, conical insecticide-treated nets or in the event that the conical shape is not readily available, rectangular shape with hanging accessories are recommended from the findings of the study.

## References

[1] World Health Organization (2007). Insecticide-treated mosquito nets: a WHO position statement. Global malaria programme. http://www.who.int/malaria/publications/insecticide-treated_materials/en/index.html.

[2] Ministry of Health and Child Welfare (2007). Zimbabwe National Health Profile. Department of Disease Prevention and Control.

[3] Roll Back Malaria (RBM) (2005). Scaling up insecticide-treated netting programmes in Africa. A strategic framework for coordinated national action. http://www.rbm.who.int/partner.

[4] Atkinson JA, Bobogare A, Fitzgerald L, Boaz L, Appleyard B, Toaliu H, Vallely A (2009). A qualitative study on the acceptability and preference of three types of long-lasting insecticide-treated bed nets in Solomon Islands: implications for malaria elimination. Malaria Journal..

[5] Fernando SD, Abeyasinghe RR (2008). Community factors affecting long-lasting impregnated mosquito net use for malaria control in Sri Lanka. Trans R Soc Trop Med Hyg..

[6] Onwujekwe O, Uzochukwu B, Ezumah N, Shu E (2005). Increasing coverage of insecticide-treated nets in rural Nigeria: implications of consumer knowledge, preferences and expenditures for malaria prevention. Malaria Journal..

[7] Maxwell CA, Rwegoshora RT, Magesa SM, Curtis CF (2006). Comparison of coverage with insecticide-treated nets in Tanzanian town and villages where nets and insecticides are either marketed or provided free of charge. Malaria Journal.

[8] Ministry of Health and Child Welfare (2008). Zimbabwe National Health Profile.

[9] Saunders M, Lewis P, Thornhill A (2009). Research methods for business students.

[10] Rowe AK, Rowe SY, Snow RW (2006). The burden of malaria mortality among African children in the year 2000. Int J Epidemiol..

[11] Das ML, Paudel IS, Niraula SR, Roy L (2007). Knowledge, attitude and practice about malaria and mosquito nets in two villages of Nepal. Indian Journal for the Practising Doctor..

[12] Pettifor A, Taylor E, Nku D (2008). Bed net ownership, use and perceptions among women seeking antenatal care in Kinshasa, Democratic Republic of Congo (DRC): Opportunities for improved maternal and child health. BMC Public Health..

[13] Stewart T, Marchand RP (2005). Factors that affect the success and failure of Insecticide Treated Net Programs for malaria control in SE Asia and the Western Pacific.

[14] Thwing J, Hochberg N, VandenEng J (2008). Insecticide-treated net ownership and usage in Niger after a nationwide integrated campaign. Tropical Medicine and International Health..

[15] Pluess B, Tanser FC, Lengeler C, Sharp BL (2010). Indoor residual spraying for preventing malaria. Cochrane Database Syst Rev..

[16] Lengeler C (2004). Insecticide-treated bed nets and curtains for preventing malaria. Cochrane Database Syst Rev.

[17] Teklehaimanoit A, Sachs JD, Curtis C (2007). Malaria control needs mass distribution of insecticidal nets. Lancet..

[18] Keating J, Mbogo CM, Mwangangi J, Nzovu JG, Gu W, Regens JL, Yan G, Githure JI, Beier JC (2005). Anopheles gambiae sl and Anopheles funestus mosquito distributions at 30 Villages along the Kenyan Coast. Journal of Medical Entomology..

[19] Sharp BL, Ridl FC, Govender D, Kuklinski J, Kleinschmidt I (2007). Malaria vector control by indoor residual insecticide spraying on the tropical island of Bioko, Equatorial Guinea. Malaria Journal..

[20] Adongo P, Kirkwood B, Kendal C (2005). How local community knowledge about malaria affects insecticide-treated net use in Northern Ghana. Tropical Medicine and International Health..

[21] Baume CA, Marin MC (2008). Gains in awareness, ownership and use of insecticide-treated nets in Nigeria, Senegal, Uganda and Zambia. Malaria Journal..

[22] Binka FN, Adongo P (1997). Acceptability and use of insecticide impregnated bednets in northern Ghana. Trop Med Int Health..

[23] Alilio M, Mwenesi H, Barat L, Payes R, Prysor-Jones S, Diara M, McGuire D, Shaw W (2007). Broken promise? Taxes and tariffs on insecticide-treated mosquito nets. American journal of Tropical Medicine and Hygiene..

[24] Onwujekwe O, Hanson K, Fox-Rushby J (2004). Inequalities in purchase of mosquito nets and willingness to pay for insecticide-treated nets in Nigeria: Challenges for malaria control interventions. Malaria Journal..

[25] Minja H (2001). Introducing insecticide treated mosquito nets in Kilombero valley in Tanzania: Social and cultural dimensions.

